# Antiretroviral treatment, government policy and economy of HIV/AIDS in Brazil: is it time for HIV cure in the country?

**DOI:** 10.1186/s12981-019-0234-2

**Published:** 2019-08-14

**Authors:** Adele S. Benzaken, Gerson F. M. Pereira, Lendel Costa, Amilcar Tanuri, André F. Santos, Marcelo A. Soares

**Affiliations:** 1 Fundação de Medicina Tropical Doutor Heitor Vieira Dourado, Manaus, Brazil; 2 Departamento de IST, AIDS e Hepatites Virais, Ministério da Saúde, Brasília, Brazil; 3 Departamento de Genética, Universidade Federal do Rio de Janeiro, Rio de Janeiro, Brazil; 4 Programa de Oncovirologia, Instituto Nacional de Câncer, Rio de Janeiro, RJ Brazil

**Keywords:** HIV, AIDS, HIV cure, Antiretroviral treatment, Brazil

## Abstract

Brazil is a low-and-middle income country (LMIC) that, despite having a large population and continental dimensions, has been able to successfully fight HIV/AIDS through a number of governmental and societal measures. These included an early response to the epidemic, the development of a universal and free public health system, incisive discussions with pharmaceutical companies to reduce antiretroviral (ARV) drug prices, investments towards the development of generic drugs and compulsory licensing of ARVs. Through such measures, Brazil is among the leading LMIC towards achieving the 90-90-90 UNAIDS goals in the years to come. In this review, we analyze Brazil’s progress throughout the HIV/AIDS epidemic to achieve state-of-the-art ARV treatment and to reduce AIDS mortality in the country. The top-quality HIV/AIDS research in Brazil towards HIV prophylactic and functional cure, the next step towards the economic sustainability of the battle against HIV, is also discussed.

## Background

Brazil is the fifth largest country in the world. It covers over half of South America and harbors almost 210 million inhabitants. This immense developing South American country was hit hard by the HIV/AIDS pandemic in the 1980s; its first AIDS case was identified in 1980. From then to June 2018, over 900,000 AIDS cases have been notified in Brazil—around 559,000 men, and 307,000 women [1]. By 2017, an estimated 866,000 people were living with HIV/AIDS within its borders [2]. The Brazilian Ministry of Health’s Department of STI, HIV/AIDS and Viral Hepatitis (DIAHV) is directly responsible for dealing with this reality—and, thus, for HIV/AIDS policy development, implementation and monitoring within Brazil’s public health system, all-encompassing Unified Health System (*Sistema Único de Saúde/SUS*).

Brazil has responded to the HIV pandemic in a number of bold and innovative ways. In 1996, Brazil was the world’s first middle-income country to offer free antiretroviral therapy (ART) to all people living with HIV (PLWHIV). In 2013, it was the third to provide all PLWHIV with ART regardless of CD4^+^ T cell counts, whereas the World Health Organization began to recommend this treatment for all 2 years later, in 2015. Presently, approximately 600,000 PLWHIV are on ART in Brazil [2].

The Brazilian *treat all* policy has driven down the number of notified HIV cases in the country over recent years and led to a recent drop in AIDS-related deaths (see below). Brazil’s *combination prevention* strategy is another crucial ingredient in this response—offering free access to an array of HIV prevention resources such as male and female condoms and lubricants; HIV self-testing; regular testing for HIV and other sexually transmitted infections (STIs); pre-exposure (PrEP) and post-exposure (PEP) prophylaxis; and harm reduction.

The HIV/AIDS epidemic in Brazil is considered stable at the national level—HIV prevalence in the general population stands at 0.4% (2017) [2]—, but is concentrated among key populations (KP) such as sex workers (SW), gay men and other men who have sex with men (MSM), transsexual people, people deprived of freedom, and people who use alcohol and other drugs (PUD). In 2018, HIV prevalence among gay men and other MSM was 18% [3]; among female SW, 5% [4]; among trans people, between 17 and 37% [5]; and among PUD, 5% [6]. KP vulnerability is heightened by a number of social and economic characteristics—such as being black, deprived of freedom or living on the streets [7].

Brazil began to offer PrEP to SW, MSM, trans people and serodiscordant couples in January 2018, based on national guidelines and on evidence from two demonstration studies. The Ministry of Health initially invested US$ 2.7 million to meet the first year’s demand of 3.6 million pills. During roll out by Brazil’s Public Health System, PrEP was dispensed—at least once—to more than 8000 people. Of the total number of people on PrEP in 2018, 83% were MSM and 41% were 18–29 years old.

## Current state of Brazil towards the UNAIDS 90-90-90 goal

**Fig. 1:**
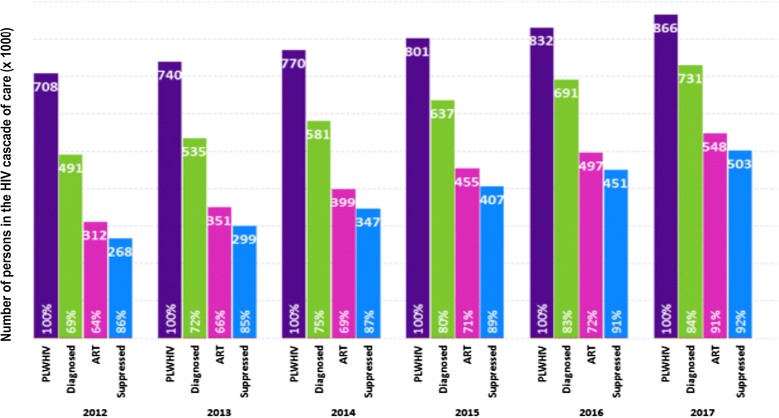
Brazil’s continuum of care cascade, 2012–2017. People leaving with HIV (PLWHIV) groups are represented by vertical bars of different colors: total persons in purple; persons diagnosed in green; persons on ART in pink; treated persons with suppressed HIV viremia in blue. Numbers at the top of each bar are in thousands. Percentages at the bottom of each bar are relative to the total number of the previous category

Brazil’s response has been progressing steadily towards achieving UNAIDS’ 90-90-90 treatment target. In 2012, there were an estimated 708,000 PLWHIV in Brazil: 69% diagnosed; 64% on ART; and 86% virally suppressed. As of 2017, the number of PLWHIV was about 866,000: 84% (731,000) diagnosed; 75% (548,000) on ART; and 92% (503,000) virally suppressed [2] (Fig. 1). Brazil reached the viral suppression 90% goal in 2015, only followed by Chile in Latin America.

**Fig. 2:**
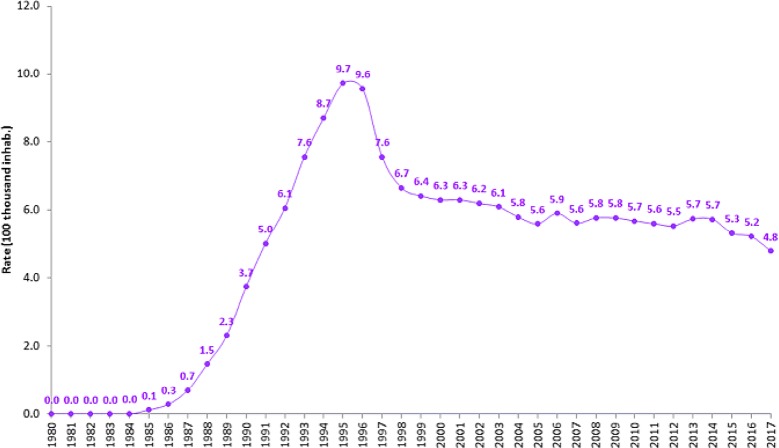
Standard AIDS mortality rates in Brazil, 1980–2017. Per year rates are represented per 100,000 inhabitants

After the implementation of treatment for all, identified cases of AIDS in Brazil have dropped from 42,184 (2012) to 37,791 (2017); and AIDS detection rates have dropped 15.7%—from 21.7 (2012) to 18.3 (2017) cases per 100,000 inhabitants. Moreover, 20 years after a significant reversal in AIDS mortality in 1996, AIDS-related deaths have recently shown a downward trend (Fig. 2). Between 2014 and 2017, a 5% drop in national AIDS-related deaths—from 5.5 (2014) to 4.8 deaths (2017) per 100 thousand inhabitants—was observed [1].

## History of antiretroviral treatment in Brazil

Antiretroviral treatment provided by Brazil’s Public Health System (SUS) has been funded entirely by national resources since its conception. In 1996, universal, free-of-charge antiretroviral therapy was established in Brazil by Law No. 9313 [*Lei Sarney*], thus ensuring the Brazilian HIV/AIDS Program’s financial sustainability. In 2007, Brazil decreed compulsory licensing of an antiretroviral drug (ARV) for the first time.

Since 2017, the country’s national preferred first-line regimen has been dolutegravir (DTG) + lamivudine (3TC) + tenofovir (TDF). The preferred second-line regimen is TDF + 3TC + Efavirenz (EFV) (as of 2014, as a three-in-one pill simplifying ART and enhancing adherence) [8]. Moreover, other ARVs in 38 different presentations are available to PLWHIV with comorbidities or virologic failure to ART, to which first- or second-line schemes are not adequate. By December 2018, around 200,000 PLWHIV were using DTG-containing regimens, and among those 57% were newly treated, and 16%, 10% and 9% were switched from EFV-, atazanavir- and raltegravir-containing regimens, respectively.

Studies conducted by the Brazil’s HIV/AIDS Program showed the superiority of DTG-containing regimens when compared to other first-line regimens. The odds ratio of failing to achieve HIV viral load suppression, with 3TC + TDF + DTG as the reference and controlling for possible confounders, vary from 1.42 for 3TC + TDF + EFV to 2.62 for 3TC + TDF + LPV/r [9]. Viral suppression was also shown to be faster among those in DTG-containing compared to EFV-containing regimens [9]. In that study, 81% of individuals that started ART with DTG-containing regimens presented viral load below 50 copies/mL after 3 months of treatment; this proportion was only 61% among those taking EFV-containing regimens. At last, it has also been shown that 3TC + TDF + DTG led to a greater CD4^+^ T-cell count recovery after the first year of treatment than did 3TC + TDF + EFV, in all CD4 strata analyzed and after controlling for possible confounders [10].

## Standard of HIV/AIDS care in Brazil

Brazil’s 2017 guidelines for HIV in adults [*Protocolo Clínico e Diretrizes Terapêuticas para o Manejo da Infecção pelo HIV em Adultos 2017*] [8] state that PLWHIV on ART should visit health services to monitor drug resistance and adherence with a frequency defined at specific situations. Those included 7 to 15 days after ART start or switch; every month/2 months until adapted to ART; every 6 months during ART, when asymptomatic and virally suppressed; case to case during ART, when symptomatic, presenting uncontrolled comorbidities and not virally suppressed; case to case, for PLWHIV who have not started ART.

HIV viral load (VL) testing also varies according to the following parameters: every 6 months during ART; 8 weeks after ART start or switch, when there is a virologic failure; or 4 weeks after first detectable VL and confirmed virologic failure [8].

In Brazil, resistance testing/genotyping is offered in cases of virologic failure after 6 months on ART. Genotyping is only recommended before ART start in women living with HIV who are pregnant, or in PLWHIV starting ART with EFV. HIV genotyoing tests are sent to a central genotyping laboratory in the city of São Paulo. Results are ready in up to 12 days and then analyzed by a team of specialized genotyping physicians in strict compliance with Brazil’s guidelines for HIV in adults [8].

## Transmitted HIV drug resistance in Brazil

One of the main challenges of maintaining undetectable HIV viral load is the emergence of drug resistance mutations (DRMs) in the viral genome under the selective pressure of ART. In some cases, treatment-naïve patients may acquire viral strains carrying DRMs, a phenomenon known as transmitted drug resistance. Some DRMs are typically found in those cases, and they are recognized as transmitted DRMs (TDRMs). The list of TDRMs from the HIV Stanford Database includes 93 resistance mutations, being 34 related to NRTI, 19 to NNRTI and 40 to PI [11]. The preconization of genotype-resistance testing in the clinical routine for the general population (every HIV-infected individual) is controversial due to its cost. In resource-rich countries, guidelines recommend the HIV-1 genotype test for the protease and reverse transcriptase viral genomic regions at the time of diagnosis to assure the success of the first line ART in viral suppression [12, 13]. The Brazilian Health Ministry recommendation for HIV genotyping covers people infected by partners under HIV treatment, pregnant women, children under 12 years-old and HIV/tuberculosis co-infected patients. In Brazil, the rates of TDR in HIV-1-infected adults have varied between 4 and 16% using Sanger sequencing of HIV RNA population from plasma [14–22] except for Amapá State, with a rate of 1% [23], similar to the lower rates (2%) found in the country in early 2000s [24]. Some studies have suggested a small, non-significant increase in the frequency of TDRM among HIV-1-infected adults in the last years, mainly to the NNRTI class, followed by NRTI and PI [14, 15], being the TDRM rates of 23% in HIV patients in the acute phase and 13–18% in patients with recent chronic infection [12, 14, 18, 25]. The clinical consequences of the presence of TDRM include a longer time to reach virological suppression and a higher risk of treatment failure [26, 27]. Although TDRM rates are considered at an intermediate level in Brazil, the recent introduction of integrase inhibitors (raltegravir, and more recently dolutegravir) in first-line ART assures a successful treatment, with 92% of patients on treatment sustaining an HIV VL below 1000 copies/ml (see above).

## The Brazilian HIV/AIDS economy

Antiretroviral drugs are distributed to PLWHIV in Brazil by 953 official ARV dispensation centers [*Unidades de Dispensação de Medicamentos*/UDM] across the country´s 5570 municipalities. Drug dispensation and stocks are monitored by an internet-based ARV logistics management system [*Sistema de Controle Logístico de Medicamentos/*SICLOM] that is hosted entirely within Brazil’s HIV/AIDS Program. Individual-level patient data is collected in 97% of all dispensation centers in Brazil and gathered information of almost 100% of PLWHIV on ART in 2018.

Presently, the budget for the Brazilian HIV/AIDS Program is approximately US$ 408 million per year; approximately US$ 302 million are for ART alone. Dolutegravir as a first-line drug was only made sustainable following intense negotiation between the Brazilian government and pharmaceutical industries, leading to a 70.5% drop in prices—from US$ 5.10 to US$ 1.50 a pill. This meant approximately US$ 139.2 million in savings for Brazil only in that first acquisition in November 2016. Currently, Brazil’s HIV/AIDS Program pays US$ 1.00 for a DTG pill.

Incentives to national generic ARV also sustained the Brazilian HIV/AIDS Program. Zidovudine (AZT) began to be locally produced in 1994. Facing the ineffectiveness of single-drug ART, SUS began to distribute the *AIDS cocktail* combination drug therapy in 1996, now simply called modern ART. Currently, eight of the 38 ARVs supplied by SUS are made in Brazil by public laboratories; private laboratories produce another five.

## HIV cure strategies: status in Brazil

Thirty-six years have passed since the beginning of the HIV-1 epidemic. Although many findings in the HIV treatment are now available, with new antiretroviral drugs and different strategies to combine them, there is still no reliable way to cure an HIV-infected individual. With the notification of the first person presumed to be cured in 2009 [28], and recently the second [29], due to a CCR5 delta32/delta32 bone marrow transplantation, the primary goal of the HIV scientific community around the globe became to uncover a reproducible and efficient strategy to cure infected individuals [30].

The establishment of HIV latency is considered one of the major obstacles to a cure strategy for HIV infection. Virus latency consists of integrated intact viral DNA with replicative competence into the host cell genome without viral production. Several factors contribute to the establishment of latency, among them the viral DNA integration site, the chromatin environment, the activation state of the infected cell and the bioavailability of cellular transcription factors. Some studies have shown the establishment of latency at early stages of HIV infection, both in humans and in non-human primate models. Latency of HIV was initially described in long-lived resting memory CD4^+^ T cells, however other cell subsets may contain latent viral clones and these may be located in diverse tissues, making it difficult to develop effective strategies for cure [31, 32]. Two approaches have been recently considered the main focus for research studies that aimed to find this cure: complete elimination of the virus in the infected patient (sterilizing cure) and establishment of immunological and molecular mechanisms in the host that prevent the latent virus from replicating again, even in the absence of cART (functional cure) [30].

Consortia of scientists work together worldwide on a variety of methods to find either a sterilizing or a functional cure for HIV infection, such as impairing viral replication through transplantation of hematopoietic cells resistant to infection [28, 29, 33, 34], gene therapies with the use of zinc finger nucleases (ZFN), transcription-activator-like effector nuclease (TALEN) or CRISPR/Cas9 [35, 36], the use of latency reversal agents (LRAs) as protein kinase C (PKC) agonists and histone deacetylase inhibitors (iHDACi) [37–41], cell death agents [42], latency silencing techniques [43], T-cell vaccines, broad spectrum neutralizing antibodies [44], and many others. In the context of Brazilian research on HIV cure, some in vitro approaches have contributed substantially to the understanding of LRA mechanism of action as well as to methods for gene editing against HIV infection. Of note, Brazilian teams in collaboration with international HIV research groups have been focusing their attention to a PKC agonist isolated from the latex of plants of the Euphorbiaceae family. Semisynthetic molecules derived from ingenol (ingenol-B), were already synthesized and had their activity evaluated on different cell models and animal studies on rhesus macaques showing promising results [40, 41]. More recently, Brazilian researchers started to explore a new promising phorbol-ester isolated from the plant *Synaderium grantii* (“Janauba”) which showed a remarkable capacity to induce HIV from a latent state in human lymphocytes in ex vivo assays [39] and further animal studies with this new molecule are already being designed.

Research focusing on the chemokine receptor gene (*CCR5*) is also well explored by Brazilian groups. CCR5 is an important co-receptor for HIV entry into the host cell and studies on this approach are already in clinical trial steps (e.g., trial # NCT02500849). The Tanuri lab had a pioneer contribution in Brazil to the CCR5 DNA editing research by disrupting the *CCR5* gene in a cellular experimental model (HEK293T epithelial cells), then evidencing the ability of the TALEN methodology to efficiently edit the genome of human cells [45]. Subsequently, with the advent of the CRISPR/Cas9 methodology, understanding the differences between the two methodologies in the *CCR5* gene edition was an important contribution to the search for more robust and reproducible DNA editing techniques in vitro [46]. Thus, the group started to work on a different strategy using CRISPR RNAs that were specific to the HIV-1 LTR promoter region but associated with a deactivated Cas9 enzyme fused to a transcriptional repressor in order to prevent viral replication in lymphocytes in vitro by CRISPR/dCas9-mediated repression.

With respect to clinical trials to reach a functional cure, a research group from Sao Paulo State (Diaz lab) organized a study to measure the impact of isolated and combined strategies in decreasing total HIV-1 DNA. They investigated the effect of treatment intensification with DTG with and without maraviroc (MVC), nicotinamide (NA; a histone deacetylase inhibitor), and auranofin. Six arms with five patients each followed every 4 weeks for a total of 48 weeks were analyzed (trial # NCT02961829). Selected patients were combinatorial ART (cART)-suppressed for > 2 years, with nadir CD4^+^ T-cell counts > 350. Groups were: (1) continuation of cART, (2) intensified cART (cART + DTG or MVC), (3) intensified cART and HDACi (cART + DTG/MVC + NA), (4) intensified cART and auranofin (cART + DTG/MVC + auranofin), (5) partially intensified cART (cART + DTG), (6) partially intensified cART (DTG) + NA + auranofin. Auranofin was used for the first 24 weeks of the study in groups 4 (G4) and 6 (G6). After week 48, a decrease in viral DNA was observed in G6 as compared to all other groups (p = 0.022; odds ratio: 9.75, 95% CI 1.1–72.39). Intensified cART with DTG + MVC presented higher decrease in the total DNA as compared to intensified cART with DTG only (G2 vs. G5, p = 0.014). All individuals presented undetectable viral loads throughout the study, but G1 showed a significant linear trend towards an increase of the viral reservoir (p < 0.05). Based on these preliminary data, the researchers suggest that NA + auranofin administration in combination with intensified ART is well tolerated, and an impact on the proviral reservoir size is possible using this combination [47]. Other studies by the same group also focused on the epigenetic factors associated with HIV latency [48] and HIV purging from latent reservoirs [49], all aiming to reduce HIV load from those compartments. Functional cure has also been approached by the Soares lab in Brazil, through the development of a multi-epitopic CTL therapeutic vaccine based on archived HIV-1 proviruses and according to prevalent HLA class I alleles in the Brazilian population. The project focuses on sequencing HIV-1 full-length proviruses archived in persons with sustained virological control with ART by next-generation sequencing (NGS), coupled to HLA A, B and C genotype determination (also by NGS). The idea will be to develop customized HIV vaccine immunogens that are archived in the patient and which are restricted by the patient´s HLA system, therefore allowing HIV control upon ART discontinuation [50].

## Conclusions

Brazil provides a state-of-the-art antiretroviral therapy, composed of the most modern ART drugs, which is largely disseminated to the HIV population through a unified, universal and free-of-charge public health system. The use of ART as a prevention tool to HIV acquisition (PrEP and PEP), coupled to a modern and efficient, decentralized HIV testing capability, all add up to an increasing number of HIV^+^ subjects who become immediate clients of the country’s health system. HIV transmitted and acquired drug resistance are also contained to low to moderate levels, resulted from the rational distribution and use of ARV drugs in the country. Despite the successful price negotiations with international pharmaceutical ARV manufacturers and the significant local production of ARVs, the costs of a universally free health system for HIV/AIDS is challenging and may be unsustainable in the long-term, urging the need of establishing approaches that progressively reduce the use of ARVs. HIV cure approaches are on the rise and Brazil already hosts several fronts of research in the field, showing promising results. Such approaches should help to alleviate the financial burden imposed on an increasing number of ARV clients (both for therapeutic and prophylactic purposes) in the country.

## Data Availability

Not applicable.
